# Nivolumab adjuvant therapy for esophageal cancer: a review based on subgroup analysis of CheckMate 577 trial

**DOI:** 10.3389/fimmu.2023.1264912

**Published:** 2023-10-04

**Authors:** Yan Lin, Huan-Wei Liang, Yang Liu, Xin-Bin Pan

**Affiliations:** ^1^ Department of Gastroenterology, Jiangbin Hospital of Guangxi Zhuang Autonomous Region, Nanning, Guangxi, China; ^2^ Department of Radiation Oncology, Guangxi Medical University Cancer Hospital, Nanning, Guangxi, China

**Keywords:** esophageal cancer, adjuvant immunotherapy, nivolumab, CheckMate 577, subgroup

## Abstract

Esophageal cancer is the sixth most common cancer worldwide. Approximately 50% of patients have locally advanced disease. The CROSS and NEOCRTEC5010 trials have demonstrated that neoadjuvant chemoradiotherapy followed by surgery is the standard treatment for patients with resectable disease. However, a pathological complete response is frequently not achieved, and most patients have a poor prognosis. The CheckMate 577 trial demonstrates that nivolumab adjuvant therapy improves disease-free survival in patents without a pathological complete response. However, there are still numerous clinical questions of concern that remain controversial based on the results of the subgroup analysis. In this review, we aim to offer constructive suggestions addressing the clinical concerns raised in the CheckMate 577 trial.

## Introduction

Esophageal cancer causes 500,000 deaths annually and stands as the sixth most prevalent cancer globally (1). Given challenges in early detection or screening, a majority of patients present with locally advanced disease. Neoadjuvant chemoradiotherapy followed by radical surgery represents the standard treatment for patients with resectable disease (2–7). Post-surgery, observation becomes the standard care protocol (4). However, patients without a pathological complete response after surgery confront a high risk of treatment failures (8–15). Unfortunately, adjuvant therapies have shown limited effectiveness (16–18). Therefore, the quest for a potent adjuvant treatment to enhance survival outcomes continues.

**Figure 1:**
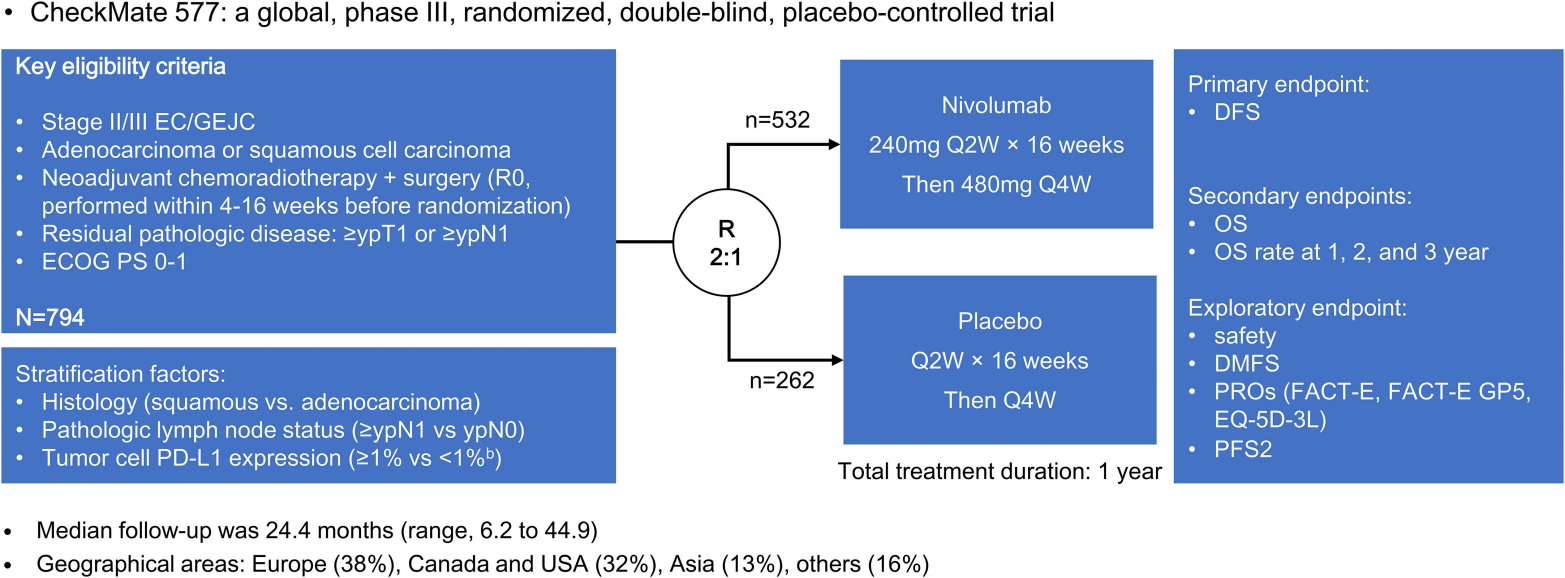
The CheckMate 577 trial design.

The CheckMate 577 trial included 794 patients with stage II or III esophageal or gastroesophageal junctional adenocarcinoma or squamous cell carcinoma (19). Patients who underwent neoadjuvant chemoradiotherapy followed by surgery without achieving a pathological complete response were randomly assigned in a 2:1 ratio to receive either nivolumab adjuvant therapy or placebo (**Figure 1**). Notably, nivolumab adjuvant therapy yielded a significant improvement in median disease-free survival compared to placebo (22.4 vs. 11.0 months; hazard ratio [HR] = 0.69, 96.4% confidence interval [CI] = 0.56-0.86; P < 0.001). This favorable trend was consistently observed across various subgroups identified through subgroup analysis.

The CheckMate 577 trial alters treatment strategies for patients with resectable esophageal cancer. However, several clinical questions of concern remain controversial. This review is conducted based on the subgroup analysis of CheckMate 577 to address perplexing questions and clinical concerns.

## Does the poor disease-free survival in the placebo group lead to statistically significant results?

**Table 1** shows the survival rates of the neoadjuvant chemoradiotherapy followed by surgery group in different studies. The median disease-free survival of the placebo group in the CheckMate 577 trial was 11.0 months (19), a result notably worse than previous studies (6, 7, 20–22). Specifically, the CROSS trial reported better outcomes, with a median disease-free survival 7 times higher in the squamous cell carcinoma subgroup and 3 times higher in the adenocarcinoma subgroup compared to the CheckMate 577 trial. Although survival time was assessed from different points [randomization before neoadjuvant chemoradiotherapy in CROSS, and randomization (4 to 16 weeks post-surgery) in CheckMate 577], it cannot fully account for the divergence between the two trials. Therefore, it is suggested that the poor disease-free survival of the placebo group may have contributed to the statistically significant results in the CheckMate 577 trial.

**Table 1:** Survivals of neoadjuvant chemoradiotherapy followed by surgery for esophageal cancer among studies.

Study	Design	Country	Cancer	Pathology	Sample	DFS/PFS		OS	
						NeoCRT+S	S	NeoCRT+S	S
CROSS	RCT	Netherlands	EC EGJC	AC	275	29.9m	17.7m	43.2m	27.1m
				SCC	84	74.7m	11.6m	81.6m	21.1m
NEOCRTEC5010	RCT	China	EC	AC	0				
				SCC	451	100.1m	41.7m	100.1m	66.5m
NeoRes I	RCT	Norway Sweden	EC EGJC	AC	65	19.5m		30.8m	
				SCC	25	49.4m		60.0m	
FFCD9901	RCT	France	EC	AC	48	27.8m	26.7m	31.8m	41.2m
				SCC	121				
Marieke et al	cohort study	Netherlands	EC EGJC	AC	527	19.6m		32.2m	
				SCC	107	21.7m		32.3m	
CheckMate 577	RCT	Global	EC EGJC	AC	187	11.1m			
				SCC	75	11.0m			

RCT, randomized controlled trial; EC, esophageal cancer; EGJC, esophagogastric junction cancer; AC, adenocarcinoma; SCC, squamous cell carcinoma; NeoCRT, neoadjuvant chemoradiotherapy; S, surgery; DFS, disease-free survival; PFS, progression-free survival; OS, overall survival.

This hypothesis aligns with a population-based study associated with the CheckMate 577 trial, where survival was assessed 12 weeks post-surgery (22). This study demonstrated better disease-free survival (19.7 months) than the CheckMate 577 trial (11.0 months). Even after matching analysis, disease-free survival remained longer compared to the CheckMate 577 trial.

This unexpected finding raises questions. Clinical trials often have strict inclusion and exclusion criteria to create a controlled and homogenous patient population. Participants are closely monitored, and their adherence to treatment protocols is carefully tracked. Therefore, there’s a strong emphasis on patients completing their treatment regimens. In contrast, in real-world settings, patients may have a wider range of characteristics, comorbidities, and complexities that can influence treatment regimens. Dose reduction and changes in treatment regimens due to the substantial systemic toxicities associated with both conventional and contemporary regimens, especially augmented regimens, have a significant impact on treatment outcomes. Therefore, in real-world studies, treatment outcomes are often suggested to be similar or worse compared with clinical trials (23–25).

A potential explanation for the poor disease-free survival of the CheckMate 577 trial may be the quality control of neoadjuvant chemoradiotherapy and surgery. The CROSS trial’s standard comprised 41.4 Gy radiotherapy plus carboplatin-paclitaxel chemotherapy. However, fewer CheckMate 577 placebo patients (63%) received ≥41.4 Gy radiation, and fewer (68%) received carboplatin-paclitaxel than in the population-based study.

Another possible explanation could be attributed to the centralization of esophageal surgery and the introduction of mandatory national surgical audits (26, 27). Prior to the centralization efforts and the implementation of surgical audits, a substantial 73.4% of patients undergoing surgical intervention received resections at low-volume hospitals (28). Furthermore, there existed substantial discrepancies in the quality of esophageal cancer care across different healthcare facilities. However, with the consolidation of resources, post-centralization, approximately 63.2% of surgically treated patients underwent resection at one of two high-volume regional centers in the Eindhoven Cancer Registry area, located in the southern part of the Netherlands (28).

These measures have demonstrably led to improvements in survival outcomes and a reduction in complications among individuals grappling with esophageal cancer. A noteworthy study assessing the treatment and survival trends among esophageal cancer patients in the Netherlands unveiled promising results (29). The 5-year relative survival rates exhibited a commendable increase, climbing from 8% to 22% for all patients with esophageal cancer. Moreover, for non-metastatic adenocarcinoma patients, the rates escalated from 12% to an encouraging 36%, while for non-metastatic squamous cell carcinoma patients, the rates surged from 9% to an impressive 27%. In tandem with these improved survival figures, a concomitant reduction in complications stemming from surgery and/or chemoradiotherapy was observed during the same period (27).

In contrast, the CheckMate 577 trial encompassed a vast network of 170 centers spanning 29 countries globally, wherein the placebo group registered an average enrollment of merely 1 to 2 patients per center. This dispersion introduced a notable variance in the quality of esophageal cancer surgery across these diverse centers. Notably, the examination of disease-free survival between the adenocarcinoma and squamous cell carcinoma subgroups yielded no significant distinction (11.1 versus 11.0 months), providing further support for the notion that inadequate quality control of chemoradiotherapy and surgery may have played a role in the diminished survival outcomes witnessed in the CheckMate 577 trial. For a comprehensive overview of the dissimilarities in treatment, refer to **Table 2**.

**Table 2:** Treatment difference between the population-based study and CheckMate 577 trial.

°C	Population-based study	CheckMate 577 trial
radiation dose(≥41.4Gy)	97%	63%
carboplatin plus paclitaxel	99.7%	68%
disease-free confirmed	12 weeks after surgery	4 weeks prior to randomization
surgery	Netherlands	170 centers in 29 countries

Nevertheless, the interpretation of the median disease-free survival of CheckMate 577 should be approached with caution. Although median disease-free survival is a commonly used endpoint in clinical trials, the timing of a select few events in the Kaplan-Meier curve can significantly influence the calculation of this median metric. In contrast, the concept of restricted mean survival time considers the entirety of the time-course of the curve, without assuming constant event risks throughout the follow-up period (30, 31). This approach provides a more pragmatic understanding of survival outcomes.

Notably, Mengato et al. recalibrated the Kaplan-Meier curves of the CheckMate 577 trial utilizing restricted mean survival time (32). Their analysis revealed that nivolumab indeed resulted in an enhanced disease-free survival when contrasted with the placebo (28.54 versus 22.70 months). Despite the conservative nature of the restricted mean survival time method, the disease-free survival remained inferior in both the nivolumab and placebo groups when compared to the CROSS trial. Consequently, it is prudent to undertake further evaluation, perhaps through the lens of overall survival, over a more extensive follow-up period to confirm the true benefits of nivolumab.

In summary, the disparities in the quality of neoadjuvant chemoradiotherapy and surgery present a plausible avenue to enhance the prognosis of esophageal cancer. The imperative of stringent quality control is as vital as the development of innovative treatment strategies within clinical practice. Moving forward, a thorough assessment of nivolumab adjuvant therapy should be undertaken in patients who have undergone surgical resection for esophageal cancer following high-quality neoadjuvant chemoradiotherapy. Such patients might avoid the need for exposure to treatment-related toxicity and its concomitant costs. In contrast, a multidisciplinary approach should be adopted for patients who have received suboptimal control in their chemoradiotherapy or surgery, in order to ascertain the suitability of nivolumab adjuvant therapy.

## Is PD-L1 expression a predictive factor?

Approximately half of individuals with esophageal cancer exhibit programmed death ligand 1 (PD-L1) expression at 1% or higher (33, 34). Elevated PD-L1 expression is correlated with improved prognosis (35, 36). Notably, a tumor cell proportion score (TPS) of ≥1% has been linked to substantial benefits in extending overall survival compared to TPS < 1%, suggesting this threshold as a potential predictor of the efficacy of PD-1/PD-L1 inhibitors (37). In the CheckMate 648 trial, the addition of nivolumab to chemotherapy enhanced overall survival in patients with TPS ≥ 1% (HR = 0.54, 99.5% CI: 0.37-0.80; P < 0.001) as well as in the overall population (HR = 0.74, 99.1% CI: 0.58-0.96; P = 0.002) (38). Conversely, in patients with TPS < 1%, nivolumab plus chemotherapy failed to yield improved overall survival (HR = 0.98, 95% CI: 0.76-1.28).

In the CheckMate 577 trial, nivolumab adjuvant therapy did not impart enhanced disease-free survival among patients with TPS ≥ 1% (HR = 0.75, 95% CI: 0.45-1.24). However, patients with TPS < 1% experienced improved disease-free survival (HR = 0.73, 95% CI: 0.57-0.92), yielding an unexpected outcome. A conceivable explanation might lie in the challenges of quantifying PD-L1 expression as a proxy for response. Crude measurements from samples could potentially underestimate true positivity, given the potential inadequacy of samples with varying levels of positivity across different tumor regions. Another factor might be that PD-L1 expression status was determined from post-surgery tumor tissue specimens in the CheckMate 577 trial, which may not accurately reflect the actual status before treatments. The relatively small sample size of patients with TPS ≥ 1% (13% in the nivolumab group and 10% in the placebo group) could also contribute to the wide confidence interval of the HR for recurrence.

Furthermore, it’s important to acknowledge the dynamic nature of PD-L1 expression during treatment. Chemoradiotherapy, for instance, can initially upregulate PD-L1 expression in tumor cells, followed by a subsequent decrease (39–42). In the CheckMate 577 trial, the interval between completing chemoradiotherapy and undergoing surgery was 4 to 6 weeks, possibly leading to 71.8% of patients exhibiting TPS < 1%. This highlights the potential insufficiency of a one-time evaluation of PD-L1 expression to predict immunotherapy efficacy (43). Therefore, assessing PD-L1 expression status before commencing chemoradiotherapy is critical for gauging its impact on prognosis.

Conversely, the combined positive score (CPS) has also emerged as a biomarker in esophageal cancer (44). Notably, anti-PD-1 treatments significantly bolstered progression-free and overall survival in the CPS ≥ 10 group in comparison to the CPS < 10 group (37, 45). Notably, anti-PD-1 treatments significantly bolstered progression-free and overall survival in the CPS ≥ 10 group in comparison to the CPS < 10 group (46). The extent of overall survival improvement was notably higher in patients with CPS ≥ 1, with no substantial enrichment at higher cut-off values. Hence, CPS > 1 may be a suitable threshold.

In the CheckMate 577 trial, patients with CPS < 5 (HR = 0.89, 95% CI: 0.65-1.22) did not witness advantages from nivolumab adjuvant therapy. In contrast, patients with CPS ≥ 5 (HR = 0.62, 95% CI: 0.46-0.83) who received nivolumab demonstrated improved disease-free survival. This finding proposes that CPS might be a more reasonable predictive factor in esophageal cancer. Nevertheless, further investigation is warranted to ascertain whether the CPS = 1 cut-off value also holds prognostic significance.

The PACIFIC trial compared durvalumab as consolidation therapy with placebo in patients with stage III non-small cell lung cancer who did not have disease progression after two or more cycles of platinum-based chemoradiotherapy (47). It confirmed the benefit of durvalumab (10 mg/kg intravenously every 2 weeks for up to 12 months) in enhancing progression-free survival regardless of PD-L1 expression prior to chemoradiotherapy (HR = 0.59, 95% CI: 0.43-0.82 for TPS < 25%, and HR = 0.41, 95% CI: 0.26-0.65 for TPS ≥ 25%). The ATTRACTION-2 trial assessed the efficacy of 3 mg/kg nivolumab intravenously every 2 weeks as the third or more line treatment in patients with advanced gastric or gastro-esophageal junction cancer who had received two or more chemotherapy regimens (48). The ATTRACTION-3 trial enrolled patients with unresectable advanced or recurrent esophageal squamous cell carcinoma who were refractory or intolerant to one previous fluoropyrimidine-based and platinum-based chemotherapy (34). These patients were randomly assigned in a 1:1 ratio to receive either 240 mg of nivolumab every 2 weeks or the investigator’s choice of chemotherapy. The results of the three trials highlighted that immunotherapy’s clinical advantage irrespective of tumor-cell PD-L1 expression. Similarly, in the CheckMate 648 trial, nivolumab’s efficacy was evident in patients with CPS > 1 and TPS > 1% (38). As such, the comparable HRs for disease recurrence between TPS < 1% and TPS ≥ 1% suggest that nivolumab adjuvant therapy exhibited consistent effectiveness regardless of tumor-cell PD-L1 expression in the CheckMate 577 trial.

## Prognostic value of ypT and ypN stage

The achievement of a pathological complete response following resection after neoadjuvant chemoradiotherapy ranges from 29% to 43% in patients with esophageal cancer (6, 7). Notably, patients who attain a pathological complete response exhibit superior 5-year overall survival compared to those without such a response (62% vs. 38%, P < 0.001) (13). Among patients without a pathological complete response, 42% to 52% of individuals manifest ypN0 status (13, 19, 22). Importantly, patients with ypN0 experience enhanced disease-free survival (HR = 2.08, 95% CI: 1.67-2.59) and overall survival (HR = 2.12, 95% CI: 1.69-2.66) compared to those with ypN+ status.

In the CheckMate 577 trial, patients with ypN0 (HR = 0.74, 95% CI: 0.51-1.06) did not derive discernible benefits from nivolumab adjuvant therapy. On the other hand, patients with ypN+ (HR = 0.67, 95% CI: 0.53-0.86) who received nivolumab demonstrated improved disease-free survival. These findings suggest that nivolumab holds greater efficacy for patients at a higher risk of distant metastasis, while its effectiveness appears diminished for those at a higher risk of local-regional recurrence. This inference is bolstered by the observation that patients with ypT3 or ypT4 status also did not gain benefits from nivolumab (HR = 0.84, 95% CI: 0.64-1.11).

The main treatment failure pattern was locoregional recurrence in patients without pathological complete response after neoadjuvant chemoradiotherapy (6, 7, 22, 49–51). Interestingly, nivolumab adjuvant therapy in the CheckMate 577 trial did not exhibit a decrease in locoregional recurrence compared to placebo (12% vs. 17%) (19). However, nivolumab did succeed in reducing distant recurrence relative to placebo (29% vs. 39%). Notably, the median distant metastasis-free survival was 28.3 months in the nivolumab group and 17.6 months in the placebo group (HR = 0.74, 95% CI: 0.60-0.92). These findings imply that the improved disease-free survival primarily stems from the diminished occurrence of distant metastasis.

This conclusion holds merit. Distant metastasis tends to emerge within the initial two years following surgery (49–51). Subsequently, the incidence of distant metastasis remains relatively stable up to ten years, as observed in the CROSS trial (50). Nivolumab demonstrated a reduction in distant metastasis following a median follow-up of 24.4 months in the CheckMate 577 trial. However, a more extended follow-up period remains necessary to evaluate overall survival differences between the nivolumab and placebo groups, particularly since the incidence of locoregional recurrence tends to rise after the initial two years (50).

Hence, it becomes pivotal to accurately select high-risk patients who are suitable candidates for nivolumab adjuvant therapy in clinical practice. Patients with ypN+ status appear to be appropriate candidates. Notably, among patients with ypN+, those with ypT+N+ status experience notably worse estimated 5-year overall survival in comparison to those with ypT0N+ status (22% vs. 47%, HR = 2.2, 95% CI: 1.6-3.0; P < 0.001) (13, 52). Furthermore, a higher ypN stage correlates with an escalating risk of death (HR = 1.3 for ypN1, HR = 2.8 for ypN2, and HR = 4.6 for ypN3, respectively). The prognostic significance of variables such as sex, race, disease stage at initial diagnosis, histology, tumor differentiation, tumor location, and time from complete resection to nivolumab adjuvant therapy should be subjected to further assessment.

## 41.4 Gy or 50.4 Gy for radiotherapy

In the CheckMate 577 trial, the distribution of radiation doses among patients was as follows: 64% in the nivolumab group and 63% in the placebo group received radiation doses ranging from 41.4 Gy to 50.4 Gy. Subgroup analyses demonstrated that nivolumab adjuvant therapy did not improve disease-free survival in the subgroups receiving doses < 41.4 Gy (HR = 0.69, 95% CI: 0.38-1.23) and > 50.4 Gy (HR = 0.72, 95% CI: 0.46-1.13). Conversely, patients receiving radiation doses between 41.4 Gy and 50.4 Gy exhibited improved disease-free survival with nivolumab (HR = 0.73, 95% CI: 0.57-0.95). It’s possible that the relatively small sample size of patients receiving doses < 41.4 Gy (11.6%) and > 50.4 Gy (19.1%) resulted in wide confidence intervals for the hazard ratio, encompassing the value of 1.

Selecting an optimal radiation dose requires a delicate balance between therapeutic benefits and potential adverse events. Across clinical trials and clinical practice, radiation doses have varied from 20 Gy to 60 Gy (6, 7, 20–22, 53). While 50.4 Gy is preferred in North America (54), 41.4 Gy is more commonly used in Asia and Europe (6, 7). However, 50.4 Gy serves as the standard dose for definitive concurrent chemoradiotherapy in patients with inoperable esophageal cancer (55–57). Notably, no significant differences in pathological complete response rates or overall survival were observed among doses of 40-41.4 Gy, 45 Gy, 50.4 Gy, and 54 Gy (58–60). Using 50.4 Gy has been associated with severe acute adverse events and unfavorable conditions for surgery (61).

Research has indicated that a biologically effective dose of 48.85 Gy is appropriate for neoadjuvant concurrent chemoradiotherapy in resectable esophageal cancer patients (62). Consequently, a dose of 41.4 Gy/23 fractions or 40 Gy/20 fractions could be a reasonable choice. Both the CROSS trial and NEOCRTEC5010 trial demonstrated that doses of 41.4 Gy/23 fractions or 40 Gy/20 fractions were linked to a 40% pathological complete response rate and a 90% R0 resection rate (6, 7). Additionally, combining radiotherapy with immunotherapy increases the risk of pneumonitis (63–65). Given that 50.4 Gy is more likely to induce pneumonitis compared to 41.4 Gy, these findings suggest that 41.4 Gy/23 fractions or 40 Gy/20 fractions should be preferred in clinical trials.

In clinical practice, an important consideration arises regarding the suitability of 41.4 Gy as a definitive dose for patients who are not candidates for surgery. This distinction underscores the concept that neoadjuvant chemoradiotherapy is a planned modality, distinct from definitive chemoradiotherapy for curative intent or conversion therapy for specific patients. Therefore, it is crucial to thoughtfully select appropriate patients.

A reasonable approach could involve oncologists designing a radiotherapy plan using a planning dose of 50.4 Gy for neoadjuvant chemoradiotherapy in esophageal cancer patients. Throughout the course of radiotherapy, the efficacy should be assessed after reaching 41.4 Gy. If the patient is deemed suitable for surgery, definitive surgery should be conducted. Post-surgery, nivolumab could be recommended if a pathological complete response is not achieved. If a patient attains a pathological complete response, observation becomes the standard of care. For patients who are not eligible for surgery, definitive chemoradiation to 50.4 Gy can be pursued as a definitive dose.

## Adjuvant nivolumab for patients with pathological complete response after neoadjuvant chemoradiotherapy

In the CheckMate 577 trial, patients who achieved a pathological complete response were excluded due to the perception that they were at low risk for recurrences. The 5-year overall survival rate for these patients was estimated to be around 47-72% (66–70). Nonetheless, a substantial proportion of these patients, ranging from approximately 17% to 39%, eventually experienced recurrences, with locoregional recurrence being the primary treatment failure pattern. Refer to **Table 3** for a summary of survival and recurrence outcomes in patients who achieved a pathological complete response following neoadjuvant chemoradiotherapy followed by surgery.

**Table 3:** Survivals and recurrences in patients with pathological complete response.

Study	Sample	Stages	Pathology	pCR	5-year OS	Recurrence		
						Total	LRR only	DM
Oppedijk et al	213	cT2-3N0-1	SCC	28%	47%	17.0%	10%	90%
			AC					
Hagen et al	188	cT2-4N0-1	SCC	33%	52%	39.0%	17%	83%
			AC					
Zanoni et al	155	cT2-4N0-1	SCC	42%	72%	16.9%	27%	73%
			AC					
Vallböhmer et al	1673	cT2-4N0-1	SCC	18%	55%	23.4%	14%	86%
			AC					
Chao et al	313	II: 46%	SCC	25%	59%	31.4%	14%	86%
		III:54%						

AC, adenocarcinoma; SCC, squamous cell carcinoma; pCR, pathological complete response; OS, overall survival; LRR, locoregional recurrence; DM, distant metastasis.

The introduction of additional systemic chemotherapy either before or after surgery does not seem to confer any discernible benefit for these patients (71, 72). Furthermore, based on the findings of the CheckMate 577 trial, nivolumab was not recommended for patients who achieved a pathological complete response after neoadjuvant chemoradiotherapy followed by surgery. Consequently, the prevailing standard of care is observation after surgery for these patients.

However, a growing body of evidence has indicated that assessing circulating tumor DNA (ctDNA) molecular residual disease (MRD) following curative-intent treatment serves as a strong predictor of recurrence across various tumor types (73–75). For patients who have achieved a pathological complete response, it is advisable to undergo MRD testing within a specific time window of 4 to 7 weeks subsequent to neoadjuvant chemoradiotherapy followed by surgery (75). Those who test negative for MRD are classified as being at low risk of recurrence, and observation is the recommended course for these individuals. Conversely, patients who test positive for MRD are considered to be at high risk of recurrence, and nivolumab is suggested as a potential therapeutic option for them.

## Adjuvant nivolumab for patients after concurrent chemoradiotherapy

For unresectable esophageal cancer, definitive concurrent chemoradiotherapy is the standard treatment (55–57). In certain cases where patients decline surgery or are unable to tolerate the associated stresses, definitive concurrent chemoradiotherapy is advised. This approach offers comparable overall survival to surgery (76, 77), but patients undergoing concurrent chemoradiotherapy often report a better quality of life (77, 78).

After concurrent chemoradiotherapy, it is associated with a local recurrence rate ranging from 40% to 60% and a 5-year overall survival of approximately 10% to 30% (79–81). Attempts have been made to enhance survival rates by implementing adjuvant chemotherapy following concurrent chemoradiotherapy, but the effectiveness of this approach has yielded inconsistent results (82–84). Hence, observation remains a commonly recommended strategy in these cases (4, 5).

The PACIFIC trial, conducted in non-small cell lung cancer patients after concurrent chemoradiotherapy, demonstrated that adjuvant immunotherapy improved progression-free survival (HR = 0.55, 95% CI: 0.45-0.68) and overall survival (HR = 0.72, 95% CI: 0.59-0.89) (47, 85). Importantly, patients without disease progression derived benefits from adjuvant immunotherapy irrespective of their PD-L1 expression status. Nonetheless, this concept remains relatively unexplored in the context of esophageal cancer, and ongoing studies (such as KEYNOTE-975 and NCT04210115) aim to elucidate whether adjuvant immunotherapy can improve survival outcomes in patients treated with definitive concurrent chemoradiotherapy.

Until the results of ongoing studies are available, it is plausible that nivolumab adjuvant therapy might enhance treatment outcomes in esophageal cancer patients without disease progression following concurrent chemoradiotherapy. However, further investigation is warranted to determine whether all such patients can equally benefit from this approach. It’s worth noting that the CheckMate 577 trial did not include patients who achieved a pathological complete response. Consequently, it seems reasonable to consider nivolumab adjuvant therapy for patients who did not achieve a pathological complete response.

The crux of this proposal hinges on the accurate assessment of pathological complete response after concurrent chemoradiotherapy. Pathological complete response rates of 29-43% have been reported in patients undergoing surgery following radiotherapy doses ranging from 41.4 to 50.4 Gy (6, 7). Similarly, patients receiving higher radiotherapy doses (50.4-60.0 Gy) in combination with concurrent chemotherapy can also achieve comparable pathological complete response rates. However, confirming the existence of pathological complete response poses a challenge. The correlation rate between clinical and pathological complete response is approximately 30% (86).

To enhance the accuracy of pathological complete response assessment, a two-step evaluation approach has proven effective (87). In the first step, primary tumor sites and suspected lesions are evaluated using esophagogastroduodenoscopy, with at least 4 random biopsies and 4 bite-on-bite biopsies performed 4-6 weeks post-concurrent chemoradiotherapy. In the second step, patients with clinical complete response in the first stage undergo a secondary clinical response evaluation 6-8 weeks later. This evaluation comprises an 18F-FDG PET-CT scan and pathological testing of any suspected areas. Patients who maintain clinical complete response in the second step are likely to have achieved pathological complete response.

## Conclusions

Improvement in survivals has been long awaited in esophageal cancer patients after surgery. The CheckMate 577 trial provides a new therapeutic strategy for these patients, which indicates that nivolumab adjuvant therapy improves disease-free survival in patents without a pathological complete response. However, the CheckMate 577 trial has room for further in-depth and extensive discussion on subgroup analysis. Further studies are needed to identify selected patients who can benefit from nivolumab adjuvant therapy. The results of these studies will be important for treatment-making and individualized precision therapy.

## Author contributions

YLin: Conceptualization, Investigation, Methodology, Resources, Writing – original draft. H-WL: Methodology, Resources, Validation, Writing – original draft. YLiu: Investigation, Validation, Writing – original draft. X-BP: Conceptualization, Writing – review & editing.
